# Polycyclic Aromatic Hydrocarbons in drinking water of Tehran, Iran

**DOI:** 10.1186/2052-336X-11-25

**Published:** 2013-08-05

**Authors:** Hamid Karyab, Masud Yunesian, Simin Nasseri, Amir Hosein Mahvi, Reza Ahmadkhaniha, Noushin Rastkari, Ramin Nabizadeh

**Affiliations:** 1 Department of Environmental Health Engineering, School of Public Health, Tehran University of Medical Sciences, Tehran, Iran; 2 Center for Water Quality Research, Institute for Environmental Research, Tehran University of Medical Sciences, Tehran, Iran; 3 Center for Air Pollution Research, Institute for Environmental Research, Tehran University of Medical Sciences, Tehran, Iran; 4 Department of Human Ecology, School of Public Health, Tehran University of Medical Sciences, Tehran, Iran

**Keywords:** Polycyclic aromatic hydrocarbons, Drinking water, Tehran

## Abstract

Distribution and seasonal variation of sixteen priority polycyclic aromatic hydrocarbons (PAHs) were investigated in the drinking water of Tehran, the capital of Iran. Detected single and total PAHs concentrations were in the range of 2.01-38.96 and 32.45-733.10 ng/L, respectively, which were quite high compared to the values recorded in other areas of the world. The average occurrence of PAHs with high molecular weights was 79.55%; for example, chrysene occurred in 60.6% of the samples, with a maximum concentration of 438.96 ng/L. In addition, mean carcinogen to non-carcinogen PAHs ratio was 63.84. Although the concentration of benzo[a]pyrene, as an indicator of water pollution to PAHs, was lower than the guideline value proposed by World Health Organization (WHO) as well as that of Iranian National Drinking Water Standards for all of the samples, the obtained results indicated that carcinogen PAHs present in the drinking water of Tehran can cause threats to human health.

## Introduction

Drinking water is one of the oldest public health issues and is associated with a multitude of health-related concerns. These concerns are derived into microbial and chemical pollutants, which are comprehensively presented in the international guidelines for drinking water quality [1]. Because of their adverse effects on human and the environment, chemical pollutants, especially xenobiotic compounds, are of foremost importance. The presence of organic pollutants, including endocrine disruptors, organophosphorous pesticides, disinfection by-product precursors, trihalomethanes (THMs), and trichloroethylene (TCE) in water resources have been widely investigated by a large number of studies [2-5]. Polycyclic aromatic hydrocarbons (PAHs) are a group of xenobiotic chemicals which are made up of carbon and hydrogen. They represent a group of contaminants with high melting and boiling points, low vapor pressure, and very low water solubility [6,7]. In the environment, they are mostly derived from anthropogenic activities. However, they can also be released into the environment through natural incomplete combustion [8]. PAHs are ubiquitous in the environment, which can be frequently found in food [9], air [10], soil [11], and sediments [12]. Additionally, they can be detected in street dust [13], rain water [14], and urban runoffs [15]. PAHs can reach water bodies mainly through dry and wet deposition, road runoff, industrial wastewater, leaching from creosote-impregnated wood, petroleum spills, and fossil fuel combustion [16-19]. They are generally teratogenic, carcinogenic, and mutagenic and may induce lung, bladder, as well as skin cancer. In addition, exposure to high levels of PAHs has been shown to produce immunosuppressive effects and is capable of causing oxidative stress during its metabolism [20-22]. The main objective of the present study was to investigate the distribution and seasonal variation of sixteen PAHs, as priority pollutants recognized by U.S. Environmental Protection Agency (EPA), in the drinking water of Tehran, the capital of Iran.

## Materials and methods

Based on drinking water supply, Tehran was divided into six districts. Four water samples were collected from each district in each season over the period from July 2011 to May 2012 (i.e. a total of 99 samples). In order to prevent unwanted reactions, samples were collected in 1000 mL amber glass bottles with Teflon lined tops. Each sample was stored in a cooler at 4°C while being transported to the laboratory. Standard solutions of sixteen PAHs (10 mg/L in acetonitrile), including naphthalene (Nap), acenaphthylene (Acy), acenaphthene (Ace), fluorene (Fl), phenanthrene (Phe), anthracene (Ant), fluoranthene (Flu), pyrene (Pyr), benzo[a]anthracene (BaA), chrysene (Chy), benzo[a]pyrene (BaP), benzo[b]fluoranthene (BbF), benzo[k]fluoranthene (BkF), dibenzo[a,h]anthracene (DahA), indeno[1,2,3-cd]pyrene (IcdP), and benzo[g,h,i]perylene (BghiP) were purchased from Supelco Company, USA. C18 extraction cartridge was purchased from Chromaband (Manchery-Nagel, Germany). A solid-phase extraction (SPE) vacuum manifold was used for concentration and purification of solvent extracts. In addition, cyclohexane, acetone, biphenyl, and methanol were of analytical-reagent grade (Merck, Germany).

Water samples were extracted using a solid phase extraction (SPE) system according to the established procedures [23]. The applied extraction method was suitable for the extraction of a wide range of analytes, as elaborated in the EPA methods 3535A [24]. To avoid adsorption of PAHs upon glassware, 5 ml of methanol was added to the samples. The solution was mixed after adding 1 μL biphenyl to methanol (1 μg/L, as internal standard). Prior to extraction, the SPE cartridge was conditioned with 5 ml of methanol under vacuum conditions, followed by 5 ml of ultra pure water. 1000 ml of the water sample was passed through the cartridge at a flow rate of 20 ml/min. After percolating all samples through the cartridge and drying the wall of the separating funnel, the cartridge was centrifuged at 2500 rpm for 10 min to remove the residential water. Then, the cartridge was dried with an air stream for 10 min, which was followed by adding 200 ml of acetone to vapor residual water. The elution was performed with 5 ml cyclohexane. The extract was dried under a gentle stream of nitrogen at 40°C. The extract was raised into the micro vial (100 micro liters) and preserved in the refrigerator until being injected into the GC/MS instrument. The PAHs extracts were analyzed by using a 3800 Varian gas chromatography coupled to a Varian Saturn 2200 mass spectrometer, equipped with a 30 m × 0.25 mm i.d. WCOT CP-Sil 8 CB column. The GC/MS operated under the following conditions: the initial column temperature was 70°C. After an initial holding time of 1 min, the temperature was programmed to rise to 300°C at a rate of 10°C/min for 30 min. The injector and detector temperatures were 250°C and 300°C, respectively. Helium was used as the carrier gas at a flow rate of 2 ml/min. Method was according to the established procedure by Li et al. (2001) and the EPA method 8270D [23,24]. PAHs concentrations were identified based on their retention times and confirmed by comparing their mass spectra with the reference library. Calibration curves were plotted at seven concentration levels from 2 to 2000 ng/L with standard solutions containing all studied PAHs. Detection limit (DL) for individual PAHs, with a signal to noise ratio of 3, ranged from 0.8 to 2 ng/L. Concentrations that were below the DLs were assigned as not determined; in such cases, half of the DL value for that substance was considered for the calculations.

## Results and discussions

The mean recovery rate for single PAHs ranged from 36.28 to 132.57% of applied concentration. The lowest recovery rates belonged to Nap (36.28%) and BghiP (47.83%), while the highest recovery rates belonged to DahA (132.57%) and BkF (117.82%) (Table 1). The concentrations of single PAHs in the distribution system ranged from not-detectable to 438.96 ng/L. As shown in Table 2, the concentration was higher than that found in Jiangsu province, China, [25], which was in the range of 0.1-10.2 ng/L. Except for Fl, Ant, Flu and Phy, all PAHs were detected in the water samples during the study period. The maximum single PAH concentrations (ng/L) were assigned to Chy (438.96), IcdP (277.51), BkF (203.75), DahA (114.61), BghiP (67.74), and Nap (63.10). Total occurrences of single PAHs were 269 times, which 79.55% was assigned to high-molecular-weight (HMW) PAHs. Chy occurred most frequently, i.e. in 60.6% of the samples, followed by Nap (46.5%) and DahA (31.3%). Results were not in agreement with those reported by Kabzinski et al. (2002), who found that the main components of PAHs mixture in the drinking water were Nap, Acy, Fl, and Ant [26].

**Table 1:** Method parameters and analytical results for PAHs components

**Compounds PAHs**	**t**_**R **_**(min)**	**Selected ions for mass spectrometry quantification**		**R**^**2 **^**in calibration**	**Mean recovery (%)**
		**Range**	**Target**		
Nap	7.99	127–129	128	0.99	36.28
Acy	11.66	151–153	152	0.99	68.14
Ace	12.08	152–154	153	0.99	82.94
Fl	13.30	165–167	166	0.99	59.10
Phe	15.60	177–179	178	0.98	112.24
Ant	17.76	177–179	178	0.98	97.03
Flu	18.60	201–203	202	0.99	74.50
Pyr	19.06	201–203	202	0.99	82.00
BaA	21.94	227–229	228	0.97	79.74
Chy	22.03	227–229	228	0.96	64.54
BbF	24.63	251–253	252	0.96	108.14
BkF	24.50	251–253	252	0.98	117.82
BaP	24.80	251–253	252	0.98	69.70
IcdP	28.70	275–277	276	0.98	80.23
DahA	28.80	275–277	276	0.98	132.57
BghiP	29.76	277–279	278	0.98	47.83

**Table 2:** Annually means concentrations of sixteen PAHs in distribution system (ng/L)

**Compounds PAHs**	**Occurrence single PAHs (%)**	**Occurrence in total detected PAHs (n,%)**	**Range (ng/L)**	**Mean (ng/L)**
Nap	46.50	46(19.1)	ND–63.1	4.6
Acy	5.05	5(2.1)	ND–3.04	-
Ace	2.02	2(0.8)	ND–2.33	-
Fl	ND^3^	ND	-	-
Phe	2.02	2(0.8)	ND–3.43	-
Ant	ND	ND	-	-
Flu	ND	ND	-	-
Pyr	ND	ND	-	-
BaA	21.20	21(8.7)	ND–34.05	2.29
Chy	60.60	60(25)	ND–438.96	27.35
BbF	18.18	18(7.5)	ND–24.39	2.15
BkF	21.20	21(8.7)	ND–203.75	11.21
BaP	11.10	11(4.6)	ND–10.77	1.33
IcdP	23.20	23(9.5)	ND–277.51	19.70
DahA	31.30	31(12.9)	ND–114.61	6.09
BghiP	28.90	28(11.6)	ND–67.74	3.24
Total PAHs	-		32.45–733.10	85.07
Concentrations as BaP^1^	-		3.14–219.59	35.60
∑Carcinogen PAHs^2^	-		6.00–575.00	38.62

^1^ Concentration of indivisuals PAH converted to BaP concentration with using Toxic Equivalency Factors.

^2^ Sum of carcinogenic PAH.

^3^ Not determined.

A broad range of the total PAHs concentrations, i.e. from 32.45 to 733.10 ng/L, was observed in different sampling points. The mean total PAHs concentration (85.07 ng/L) was comparable with that found in Helsinki, i.e. 150.3 ng/L, and Horsholm, i.e. 106.5 ng/L [27]. However, it was lower than the concentrations detected in Kaoshing, i.e. 1452.9 ng/L [28], and Meet Faris, i.e. 1127 ng/L [29]. Badawy and Emababy evaluated PAHs distribution in the drinking water of four cities in Egypt and found that total PAHs fluctuated in the range of 703–1238 ng/L, which 80% belonged to 4-, 5-, and 6-ring PAHs. Their latest results were consistent with those observed in the present study, which demonstrated that the contribution of HMW PAHs was 79.55%.

Toxic equivalency factor (TEF) was used to evaluate single PAHs concentrations as BaP equivalent. TEF is an estimate of the relative toxicity of a PAH compared to that of Bap [7]. Results demonstrated that the mean PAHs concentration as BaP equivalent was in the range of 3.14-219.59 ng/L in the water samples. The carcinogenic PAHs concentration, including BaA, BbF, BkF, Chy, BaP, DBahA, and IcdP, which are probable human carcinogens according to the U.S. EPA (2002), were identified in the drinking water samples [30]. Sum of carcinogen PAHs ranged from 6.00 to 575.00 ng/L in various seasons. The maximum concentration of carcinogen PAHs was observed in summer, which was similar to the results from the study of Kabzinski et al. (2002). Carcinogen to non-carcinogen PAHs ratios varied from 8.12 to 98.48%, with an average of 63.84%. Detected concentrations of BaP, a carcinogen PAHs, ranged between 4.28 to 10.77 ng/L in summer and autumn, which is lower than the guideline values proposed by WHO [31] as well as that of Iranian National Drinking Water Standards [32]. However, in one sample, BaP concentration was recorded to be higher than the recommended value of European Union. The allowable level of PAHs in European Union’s drinking water standard is 10 ng/L for BaP and 100 ng/L for carcinogen PAHs [33]. In addition, the concentrations of carcinogen PAHs in 12 samples were higher than European Union’s drinking water standards.

**Figure 1:**
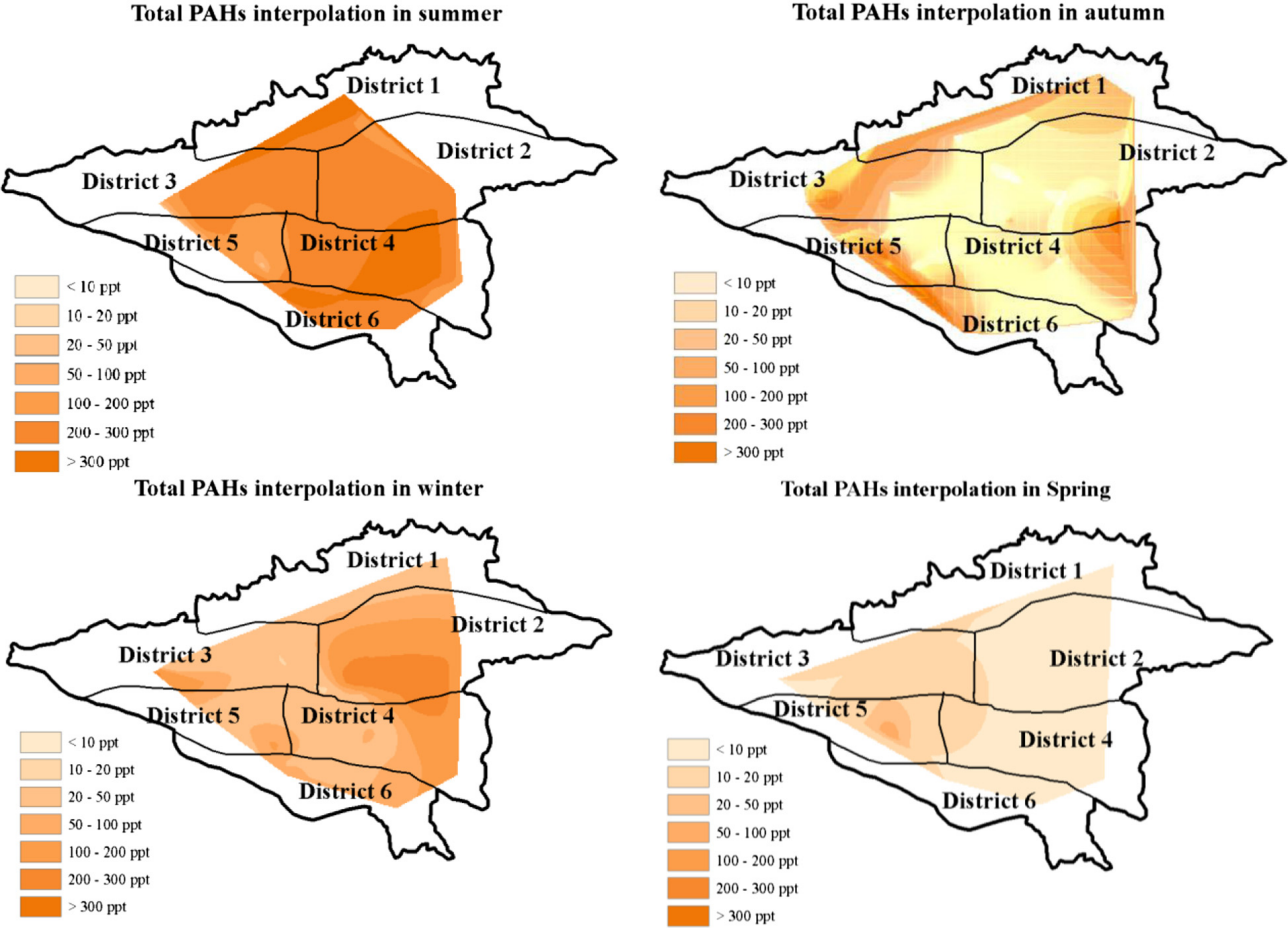
Interpolation of the spatial distribution of total PAHs in different districts.

The concentration of the single PAHs and total PAHs in the drinking water shows important variations depending on the seasonal variations. The highest concentrations of total PAHs were detected in summer, which ranged between 22.31 to 733.10 ng/L, with an average of 277.35 ng/L. Our results were lower than those detected by Kabzinski et al. (2002). They found that total PAHs concentration ranged from 38 to 4953 ng/L. In addition, they demonstrated that the highest PAHs contents took place in July–September, which was in agreement with our results. To present seasonal variation of total PAHs concentration, interpolation of pollutants in the distribution system was achieved with the natural neighbor method, the commonly used interpolation method in ARC GIS 9.2 (Figure 1). In this method, weights are computed based on the areas rather than distances from the surrounding points [34].

**Figure 2:**
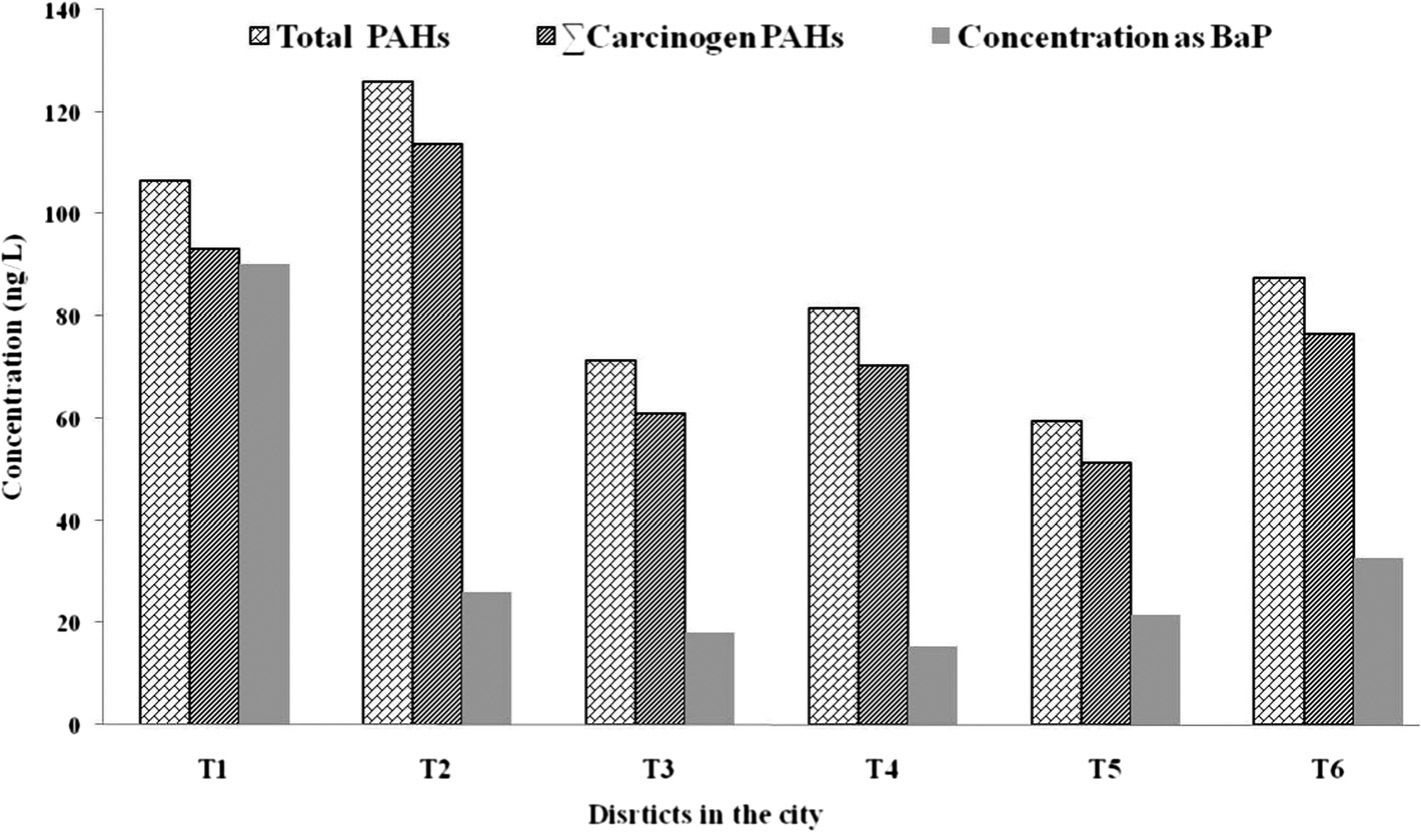
PAHs components concentration in different districts in tap water (ng/L).

Result indicated that the concentration of total PAHs in the drinking water of Tehran varies according to the geographical location. The maximum PAHs concentration was observed in district T2 (Figure 2). The main reason for PAHs presence in the distribution system can be the pollution of water sources as well as leaching from pipelines [7]. Trend of PAHs variations in the distribution system, except for winter, was comparable with PAHs concentrations in water resources. It was suggested that the highest PAHs concentration in Karaj River, the main source of drinking water for Tehran, was in summer (an unpublished observation). Comparison of PAHs concentrations in water sources and those present in the distribution system suggested that there existed significant similarity between them. In Tehran Water Treatment Plant, drinking water passes through conventional treatment operations, including coagulation, flocculation, sedimentation, sand filtration, and disinfections. Removal efficiencies of PAHs by conventional treatment processes are reported to be in the range of 20-100% [29,35]. Therefore, the conventional water treatment processes are not efficient for the removal of PAHs from drinking water supplies, and water sources can be an important route for drinking water pollution to PAHs. The main sources of PAH contamination in drinking water are rarely the raw water source; rather, the coating of the drinking water distribution pipes with coal tar is mainly responsible [7]. Coal tar is used to give effective protection against corrosion. Water disinfection by chlorine and anaerobic condition remobilized PAHs from the coal tar lining [36,37].

## Conclusion

• The first integrated investigation of PAHs in the drinking water of Tehran revealed that some individual HMW PAHs, such as Chy, BkF, and IcdP, are present in levels higher than that of European Union’s drinking water standard, whereas, the permissible level for PAHs in drinking water by WHO and Iranian National Drinking Water Standards is only set for BaP.

• In the previous studies, several organic pollutants, including THMs and halo acetic acids, were identified in Tehran water sources [38-40]. Detection of PAHs in the present study shows that there are serious pollutants in water sources; it also indicates the inefficiency of water resources management in Tehran.

• The PAH profiles in the distribution system were similar to those of surface water observed in Karaj river, which is the important source of drinking water for Tehran. The high PAHs concentrations in the summer may be the result of a high concentration of PAHs in water sources, which was observed in another part of this study (unpublished observations).

• In all sampling points, the concentration of BaP in the drinking water was lower than 700 ng/L, as recommended by WHO [31] and Iranian National Drinking Water Standards [32]. However, the concentrations of carcinogen PAHs in 12% of samples were higher than European Union’s drinking water standard, which forces that the total concentration of PAHs should not exceed 100 ng/L. In Table 3 results of detected PAHs is compared with national and international standards.

**Table 3:** Comparing the results and PAHs standards in drinking water (ng/L)

**Components**	**USEPA **[20]	**WHO **[31]	**ISIRI **[32]	**EU **[33]	**Results**
BaP	200	700	700	10	ND – 10.77
					(mean: 1.33)
Sum of BaP, BbF, BkF, Chy and DBahA	200	-	-	-	5.21 – 472.47
					(mean: 48.12)
Sum of BbF, BkF, BghiP and IcdP	-	-	-	100	4.06 – 569.72
					(mean: 36.30)

• To protect drinking water sources as well as to prevent adverse effects on humans and biota, authors’ recommendations are as below:

1. Full protection of water sources, including suppression of commercial, residential, and recreational activities in the vicinity of rivers and dams;

2. To establish national as well as international standards for permissible levels of individual polycyclic aromatic hydrocarbons, especially carcinogen PAHs;

3. To remove PAHs from water sources by advanced water treatment technologies; also, to prevent chlorinated-PAHs formation in the drinking water during chlorination [41,42].

## Competing interests

The authors declare that they have no competing interests.

## Authors’ contributions

Authors contributed to the article as follows: MY supervised the study. He was involved in study design and interpretation of results. HK was responsible for sample collection, data analysis, summarization of results and manuscript preparation. RA was responsible for analysis of PAHs in water samples collected in this study. SN, AHM and RN were participated in designing the field studies, data processing and analysis. They gave general support in interpretation of results and manuscript preparation. All authors read and approved the final manuscript.
